# Cost-effectiveness of sofosbuvir-based treatments for chronic hepatitis C in the US

**DOI:** 10.1186/s12876-015-0320-4

**Published:** 2015-08-05

**Authors:** Sai Zhang, Nathaniel D. Bastian, Paul M. Griffin

**Affiliations:** 1 School of Industrial and Systems Engineering, Georgia Institute of Technology, 755 Ferst Dr, Atlanta, GA 30332-0205 USA; 2 Department of Industrial Engineering, Penn State University, University Park, PA 16803 USA

**Keywords:** Cost-effectiveness, Markov model, Sofosbuvir, Harvoni, Olysio, Viekira Pak, Chronic hepatitis C

## Abstract

**Background:**

The standard care of treatment of interferon plus ribavirin (plus protease inhibitor for genotype 1) are effective in 50 % to 70 % of patients with CHC. Several new treatments including Harvoni, Olysio + Sovaldi, Viekira Pak, Sofosbuvir-based regimens characterized with potent inhibitors have been approved by the Food and Drug Administration (FDA) providing more options for CHC patients. Trials have shown that the new treatments increased the rate to 80 % to 95 %, though with a substantial increase in cost. In particular, current market pricing of a 12-week course of sofosbuvir is approximately US$84,000. We determine the cost-effectiveness of new treatments in comparison with the standard care of treatments.

**Methods:**

A Markov simulation model of CHC disease progression is used to evaluate the cost-effectiveness of different treatment strategies based on genotype. The model calculates the expected lifetime medical costs and quality adjusted life years (QALYs) of hypothetical cohorts of identical patients receiving certain treatments. For genotype 1, we compare: (1) peginterferon + ribavirin + telaprevir for 12 weeks, followed by 12 or 24 weeks treatment of peginterferon + ribavirin dependent on HCV RNA level at week 12; (2) Harvoni treatment, 12 weeks; (3) Olysio + Sovaldi, 12 weeks for patients without cirrhosis, 24 weeks for patients with cirrhosis; (4) Viekira Pak + ribavirin, 12 weeks for patients without cirrhosis, 24 weeks for patients with cirrhosis; (5) sofosbuvir + peginterferon + ribavirin, 12 weeks for patients with or without cirrhosis. For genotypes 2 and 3, treatment strategies include: (1) peginterferon + ribavirin, 24 weeks for treatment-naïve patients; (2) sofosbuvir + ribavirin, 12 weeks for patients with genotype 2, 24 weeks for genotype 3; (3) peginterferon + ribavirin as initial treatment, 24 weeks for patients with genotype 2/3, follow-up treatment with sofosbuvir + ribavirin for 12/16 weeks are performed on non-responders and relapsers.

**Results:**

Viekira Pak is cost-effective for genotype 1 patients without cirrhosis, whereas Harvoni is cost-effective for genotype 1 patients with cirrhosis. Sofosbuvir-based treatments for genotype 1 in general are not cost-effective due to its substantial high costs. Two-phase treatments with 12-week and 16-week follow-ups are cost-effective for genotype 3 patients and for genotype 2 patients with cirrhosis. The results were shown to be robust over a broad range of parameter values through sensitivity analysis.

**Conclusions:**

For genotype 1, sofosbuvir-based treatments are not cost-effective compared to Viekira Pak and Harvoni, although a 30 % reduction in sofosbuvir price would change this result. Sofosbuvir + ribavirin are cost-effective as second-phase treatments following peginterferon + ribavirin initial treatment for genotypes 2 and 3. However, there is limited data on sofosbuvir-involved treatment, and the results obtained in this study must be interpreted within the model assumptions.

## Background

Chronic hepatitis C (CHC) is the leading cause of chronic liver disease and the primary reason for liver transplantation [1, 2]. Approximately 170 million people worldwide are infected with the hepatitis C virus (HCV), including 4 million people in the US [3, 4]. CHC can go undetected for years, and once the symptoms do appear, liver damage has begun [5]. Approximately 42 % of CHC patients will develop cirrhosis in their lifetime [6]. Further, 23 % of these patients, if untreated, will eventually develop hepatocellular carcinoma, the primary cause of liver disease induced mortality [7]. In advanced stages of cirrhosis, liver transplantation is typically the only treatment option [8].

In the last few years, the standard of care for untreated CHC patients has changed from dual therapy with peginterfeon and ribavirin to triple treatment with peginterferon, ribavirin plus protease inhibitors (PI) such as telaprevir or boceprevir [9]. Although fairly effective compared to the older dual therapy, this triple therapy does not achieve more than a 75 % sustained virologic response (SVR) [10], which is defined as HCV RNA less than lower limit of quantification (LLOQ) at 12 weeks after the end of treatment. Once SVR is achieved, relapse is very unlikely. However, injected interferon can lead to severe side effects such as fatigue, depression, and emotional liability [2].

In December 2013, sofosbuvir (brand name Sovaldi) as a new component of interferon-free oral regimen was approved by the U.S. Food and Drug Administration (FDA) for treating CHC. The drug eliminates the need for some patients to take interferon, specifically patients with genotypes 2 and 3 [11]. These patients can use sofosbuvir alone with ribavirin, whereas patients with genotype 1 are recommended to take sofosbuvir in combination with peginterferon and ribavirin [11]. More recently, there have appeared a number of potent inhibitors that were approved as an all-oral regimen to treat genotype 1 (Table 1). In October 2014, the combination of ledpasvir-sofosbuvir (Harvoni) was approved by the FDA for the treatment of genotype 1 CHC patients with or without cirrhosis [12]. One month later, the use of simeprevir (brand name Olysio) in combination with sofosbuvir was also approved for genotype 1 patients [13]. A month later, Viekira Pak comprised of four medications (ombitasvir, paritaprevir, ritonavir and dasabuvir) was approved for genotype 1 patients as well [14]. These new treatments are characterized by significant increases in SVR [15]. The traditional regimen of peginterferon plus ribavirin is effective in 50 % to 70 % of patients with CHC. These new regimens as combinations of inhibitors increased the effective rate to 80 % to 95 % [12–14, 16–18]. However, as a popular component of new treatments, current market pricing of a 12-week course of sofosbuvir alone costs roughly $84,000 [19, 20]. We determine the cost-effectiveness of sofosbuvir-involved treatments in comparison with interferon-based treatments. To date, such analysis has not been reported, except for a recent study that found sofosbuvir-based treatments to be cost-effective for incarcerated persons [21].

**Table 1:** FDA recommendations [19]

Genotype	Treatment	Duration (weeks)
1	Harvoni	12
	Olysio + Sovaldi with or without Ribavirin	12 (no cirrhosis), 24 (cirrhosis)
	Viekira Pak + Ribavirin	12 (no cirrhosis), 24 (cirrhosis)
	Sovaldi + Peginterferon + Ribavirin	12
2	Sovaldi + Ribavirin	12
3	Sovaldi + Ribavirin	24

## Methods

We apply a Markov simulation model of CHC disease progression to evaluate the cost-effectiveness of different treatment strategies for CHC. The model calculates the expected lifetime medical costs and quality adjusted life years (QALYs) of hypothetical cohorts of identical patients receiving certain treatments. Treatments are compared based on the ratio of the additional cost of the more costly treatment divided by the additional effectiveness of the treatment. Reference patient cohorts are defined according to the average characteristics, gender and age, obtained from the trials used in this study (52-years old, 64 % male, treatment-naïve who have CHC with or without cirrhosis).

At the beginning of a period, each hypothetical patient receives a designated treatment. If the patient shows detectable HCV RNA by a PCR test throughout the therapy, the patient is classified as a non-responder. If a patient is HCV negative during therapy and also negative in the test 12 weeks after treatment, we assume SVR is achieved. Otherwise, a relapse occurs. Whether a non-responder or relapser will receive follow-up treatment depends on the specific treatment plan. After receiving treatment, each patient enters a Markov process based on the viral response result. Subsequent long-term prognosis of each treatment group is then estimated using simulation, and the cohort is tracked as a patient moves through different health states until death. Transitions are made annually based on natural disease progression and each year patients may remain in the same state or transit to another state. In accordance with the literature, [6, 22–25] the costs and benefits are discounted at an annual rate of 3 %. All costs are adjusted to 2014 U.S. dollars.

### Efficacy rates of treatments

Efficacy data associated with sofosbuvir-based new treatments are extracted from five clinical studies [16–18]. These studies include a total of 1724 HCV mono-infected patients with genotypes 1 to 6 CHC. It should be noted that these five trials targeted different patient cohorts. The primary ending point was SVR at 12 weeks after the end of treatment. In the NEUTRINO study, [17] a 12-week treatment was evaluated with Sovaldi + peginterferon-alpha + ribavirin in treatment-naïve subjects with genotype 1,4,5,6. In the study, 90 % of patients had a SVR with 89 % for patients with genotype 1. The SVR rate was 92 % among patients without cirrhosis and 80 % among those with cirrhosis. Aiming at treatment-naïve subjects with genotypes 2 and 3, the FISSON study compared 12-week treatment with Sovaldi and ribavirin to a 24-week treatment with peginterferon-alpha plus ribavirin [17]. SVR categorized by genotype and cirrhosis is shown in Tables 2 and 3. The FUSION study conducted experiments on patients previously treated with interferon with genotypes 2 or 3 who either relapsed or failed to respond, and a 12- or 16-week treatment with Sovaldi and ribavirin was performed [16]. The VALENCE trial showed that for treatment-naïve genotype 3 patients, Sovaldi plus ribavirin for 24-week treatment obtained a 93 % SVR with no cirrhosis and a 92 % SVR with cirrhosis [18]. For Harvoni treatment, phase 3 studies (ION-1,ION-2,ION-3) have consistently shown SVR rates greater than 90 % with a 12-week course in patients of genotype 1 CHC with or without cirrhosis [12]. According to the COSMOS study, SVR rates were 95 % for non-cirrhotic patients with 12-week treatment of Olysio + Sovaldi and 100 % for cirrhotic patients with 24-week treatment [13]. Viekira Pak + ribavirin regimens were characterized with 95 % SVR for non-cirrhotic patients with 12-week treatment (SAPPHIRE-I study) and 95 % for cirrhotic patients with 24-week treatment (TURQOUISE study) [14]. In all of these trials, treatments were not guided by subjects’ HCV RNA levels, implying that a no response-guided algorithm was used.

**Table 2:** Response rates for patients without cirrhosis

Genotype	Treatment	Duration (weeks)	SVR (%)	Trials
1	Telaprevir + Peginterferon-alpha + Ribavirin	12 + 12 or 12 + 36	75	ADVANCE
	Harvoni	12	98	ION
	Olysio + Sovaldi	12	95	COSMOS
	Viekira Pak + Ribavirin	12	96	SAPPHIRE
	Sofosbuvir + Peginterferon + Ribavirin	12	92	NEUTRINO
2	Peginterferon + Ribavirin (Treatment-naïve)	24	81	FISSION
	Sofosbuvir + Ribavirin (Treatment-naïve)	12	97	FISSION
	Sofosbuvir + Ribavirin (Non-responders & Relapsers)	12	90	FUSION
		16	92	FUSION
3	Peginterferon + Ribavirin (Treatment-naïve)	24	81	FISSION
	Sofosbuvir + Ribavirin (Treatment-naïve)	24	93	VALENCE
	Sofosbuvir + Ribavirin (Non-responders & Relapsers)	12	37	FUSION
		16	63	FUSION

**Table 3:** Response rates for patients with cirrhosis

Genotype	Treatment	Duration (weeks)	SVR (%)	Trials
1	Telaprevir + Peginterferon-alpha + Ribavirin	12 + 12 or 12 + 36	75	ADVANCE
	Harvoni	12	98	ION
	Olysio + Sovaldi	24	100	COSMOS
	Viekira Pak + Ribavirin	24	95	TURQOUISE
	Sofosbuvir + Peginterferon + Ribavirin (Treatment-naïve)	12	80	NEUTRINO
2	Peginterferon + Ribavirin (Treatment-naïve)	24	62	FISSION
	Sofosbuvir + Ribavirin (Treatment-naïve)	12	83	FISSION
	Sofosbuvir + Ribavirin (Non-responders & Relapsers)	12	60	FUSION
		16	78	FUSION
3	Peginterferon + Ribavirin (Treatment-naïve)	24	30	FISSION
	Sofosbuvir + Ribavirin (Treatment-naïve)	24	92	FISSION
	Sofosbuvir + Ribavirin (Non-responders & Relapsers)	12	19	FUSION
		16	61	FUSION

As benchmarks, we consider pegylated interferon, ribavirin plus telaprevir therapy as the standard care for genotype 1 and pegylated interferon plus ribavirin as the standard care for genotypes 2 and 3. They are commonly accepted treatments and acknowledged to be cost-effective in previous literature. Telaprevir is given with peginterferon and ribavirin for the first 12 weeks of therapy, followed by an additional 12 or 36 weeks of peginterferon and ribavirin depending on the response during therapy. If HCV RNA levels are undetectable at week 12 of treatment, an additional 12 weeks of peginterferon and ribavirin should be received, otherwise an additional 24 weeks of peginterferon and ribavirin are expected. As sofosbuvir also works for relapsers and non-responders with genotypes 2 and 3, we design a follow-up treatment of sofosbuvir plus ribavirin for patients who experience prior interferon treatment failure. We assume that all patients who participate in sofosbuvir-involved therapy either as initial or follow-up treatment complete the whole course of treatment. Subsequent prognosis of patients who relapse after the whole treatment is assumed to be identical to those who never have treatment.

In this study, we discuss treatment strategies based on genotypes. For genotype 1, the following treatment strategies are compared: (1) peginterferon + ribavirin + telaprevir for 12 weeks, followed by an additional 12 or 24 weeks treatment of peginterferon + ribavirin dependent on HCV RNA level at week 12; (2) Harvoni treatment, 12 weeks; (3) Olysio + Sovaldi, 12 weeks for patients without cirrhosis, 24 weeks for patients with cirrhosis; (4) Viekira Pak + ribavirin, 12 weeks for patients without cirrhosis, 24 weeks for patients with cirrhosis; (5) sofosbuvir + peginterferon + ribavirin, 12 weeks for patients with or without cirrhosis. For genotypes 2 and 3, treatment strategies include: (1) peginterferon + ribavirin, 24 weeks for treatment-naïve patients; (2) sofosbuvir + ribavirin, 12 weeks for patients with genotype 2, 24 weeks for genotype 3; (3) peginterferon + ribavirin as initial treatment, 24 weeks for patients with genotype 2/3, follow-up treatment with sofosbuvir + ribavirin for 12/16 weeks are performed on non-responders and relapsers. We do not explicitly consider the case of patients where interferon containing therapy is not an option because of contraindications or because interferon based therapy has failed.

The Markov simulation model includes the following CHC associated health states: CHC, treatment induced cure, and liver disease induced death [5, 6]. In addition, patients at each health state are subject to the same age-dependent other cause induced death rate. Transition occurs annually and depends on health state-specific transition probabilities. Due to a lack of well-designed studies of patients with CHC, transition probabilities are estimated from the most widely quoted published data. Age-dependent death rates are obtained from the 2008 United States life table [26].

### Health-state related quality adjusted life years

Quality of life specific to different health states are adjusted on an annual scale from 1 (perfect health) to 0 (death). Estimates of utilities were based on actual patients’ utilities using the health utility index [27]. To estimate treatment-specific QALYs, the time spent in each health state was multiplied by each utility value and then summed over the life expectancy. As interferon based therapy has significant side effects, a 9 % reduction in utility is assumed for interferon-based therapy [22, 25]. Since most side effects are significantly more common in interferon containing regimens as compared to interferon-free ones, we assume that adding sofosbuvir in a regimen does not change the QALY values.

### Medical costs

Medical costs are summarized in Tables 4 and 5. Drug costs are estimated using approximate 2014 average wholesale acquisition prices [12–14, 20]. Other therapy related costs including screening, diagnostic and laboratory testing, drugs, monitoring costs during therapy and follow-up periods are estimated from the literature. Annual costs associated with each health state have been previously discussed and are inflated to 2014 US dollars using the medical care component of the Consumer Price Index [28].

**Table 4:** Annual costs of care

	Annual costs of care (2014 US$)	Reference
Chronic Hepatitis C	$572.69	[25]
Compensated Cirrhosis	$762.99	[25]
Decompensated Cirrhosis	$39,675.48	[25]
HCC	$25,862.67	[25]
Liver Transplant (1st year)	$483,057.01	[25]
Liver Transplant (successive year)	$46,515.46	[25]

**Table 5:** Weekly cost of treatments

Treatment	Cost per week (2014 US$)
Harvoni	$7,875.00
Olysio + Sovaldi	$12,500.00
Viekira Pak + Ribavirin	$7,000.00
Ribavirin	$250
Peginterferon + Ribavirin	$750
Sofosbuvir	$7,000

### Sensitivity analysis

In order to evaluate the robustness of the model, sensitivity analysis is performed for all parameters. Specifically, a 95 % confidence interval is used for each entry of utility weights and natural history transition probabilities. Costs are halved and doubled. In terms of response rate, the model is reanalyzed for ±10 % change of the value for each efficacy. A variable is considered to be potentially influential if it leads to the change of effectiveness for a treatment. In addition to one-way sensitivity analyses for all variables, we conduct probabilistic sensitivity analyses. It is based on Monte Carlo simulation with 1000 runs, where parameters are varied randomly according to associated distributions. This approach examines the effect of joint uncertainty in the model’s variables. We assume that transition probabilities and utilities follow a uniform distribution with ranges specified in Tables 6 and 7. Treatment efficacies follow a Beta distribution and costs follow a Gamma distribution, all with a standard deviation equal to 10 % of the baseline value.

**Table 6:** Annual transitions (stated as percentages)

Transition	Baseline	Range	Reference
Chronic Hepatitis C			
to Compensated Cirrhosis	7.30 %	1.0 %-23.2 %	[6, 25]
Compensated Cirrhosis			
to Decompensated Cirrhosis	3.90 %	2.0 %-8.3 %	[22, 25, 29]
to HCC	3.70 %	1.0 %-4.4 %	[25, 30, 31]
Decompensated Cirrhosis			
to HCC	3.70 %	1.0 %-4.4 %	[25, 29, 30]
to Liver Transplant	3 %	1.0 %-6.2 %	[22, 25]
to Liver-induced Death	12.90 %	6.5 %-19.3 %	[15, 22, 24, 29]
HCC			
to Liver Transplant	3 %	1.0-6.2 %	[22, 25]
to Liver-induced Death	42.70 %	33 %-86 %	[25, 29]
Liver Transplant			
to Liver-induced Death, first year	13.70 %	6 %-42 %	[25, 32]
to Liver-induced Death, successive year	5.20 %	2.4 %-11 %	[25, 32]

**Table 7:** Health-state specific QALYs

QALYs	Baseline	Range	Reference
Uninfected	1	1	[27]
Chronic Hepatitis C	0.82	0.6-0.9	[27]
Compensated Cirrhosis	0.78	0.5-0.9	[27]
Decompensated Cirrhosis	0.65	0.3-0.88	[27]
HCC	0.25	0.1-0.5	[27]
Liver Transplant (1st year)	0.5	0.11-0.7	[27]
Liver Transplant (successive year)	0.7	0.24-0.87	[27]

## Results

### Base case analysis

Treatments aimed at the same group of patients (genotype, existence of cirrhosis) are compared. We consider results categorized by whether cirrhosis exists. The results for patients without cirrhosis are shown in Table 8, and the results for patients with cirrhosis are shown in Table 9. Note that the treatments are sorted according to cost in ascending order. In both tables, the incremental cost-effectiveness ratio (ICER) is calculated as the additional cost divided by additional effectiveness between each treatment and the benchmark treatment. Specifically, all treatments are first compared to the standard of care treatment (e.g., standard of care treatment for genotype 1 is Telaprevir + Peginterferon + Ribavirin), and then compared to the adjacent efficient treatment. For example, in Table 8 for genotype 2, the Adjacent ICER between two-phase treatment with 24 + 12 versus Peginterferon + Riavirin is 4233. The Adjacent ICER between two-phase treatment with 24 + 16 versus two-phase treatment with 24 + 12 is 44,458. The Adjacent ICER between Sofobuvir + Ribavirin versus two-phase treatment with 24 + 16 is 1,805,952.

**Table 8:** Base case results for patients without cirrhosis

Genotype	Treatment	Duration (weeks)	Cost ($)	Effectiveness (QALYs)	ICER compared to benchmark ($/QALY)	Adjacent ICER ($/QALY)
1	Viekira Pak + Ribavirin	12	97,380	19.9659	dominant	
	Harvoni	12	106,830	19.9618	dominant	dominated
	Telaprevir + Peginterferon + Ribavirin for first 12 weeks, followed by additional 12 or 36 weeks of Peginterferon + Ribavirin	12 + 12 or 36	108,820	18.3364	benchmark	dominated
	Sofosbuvir + Peginterferon + Ribavirin (Treatment-naïve)	12	111,790	19.568	2,412	dominated
	Olysio + Sovaldi	12	165,220	19.9356	35,268	dominated
2	Peginterferon + Ribavirin (Treatment-naïve)	24	45,560	18.7853	benchmark	
	Initial: Peginterferon + Ribavirin; Follow-up: Sofosbuvir + Ribavirin for non-responders and relapsers.	24 + 12	50,340	19.9145	4,233	4,233
		24 + 16	54,030	19.9975	6,987	44,457
	Sofosbuvir + Ribavirin (Treatment-naïve)	12	99,540	20.0227	43,624	1,805,952
3	Peginterferon + Ribavirin (Treatment-naïve)	24	52,810	18.2828	benchmark	
	Initial: Peginterferon + Ribavirin; Follow-up: Sofosbuvir + Ribavirin for non-responders and relapsers.	24 + 12	69,390	18.9351	25,418	25,418
		24 + 16	70,220	19.4892	14,431	1,498
	Sofosbuvir + Ribavirin (Treatment-naïve)	24	187,880	19.8033	88,833	374,594

**Table 9:** Base case results for patients with cirrhosis

Genotype	Treatment	Duration (weeks)	Cost ($)	Effectiveness (QALYs)	ICER compared to benchmark ($/QALY)	Adjacent ICER ($/QALY)
1	Harvoni	12	108,000	19.9787	dominant	
	Telaprevir + Peginterferon + Ribavirin for first 12 weeks, followed by additional 12 or 36 weeks of Peginterferon + Ribavirin	12 + 12 or 36	122,420	17.2075	benchmark	dominated
	Sofosbuvir + Peginterferon + Ribavirin (Treatment-naïve)	12	130,750	17.8475	13,016	dominated
	Viekira Pak + Ribavirin	24	186,820	19.7603	25,227	dominated
	Olysio + Sovaldi	24	313,310	20.136	65,184	1,305,213
2	Peginterferon + Ribavirin (Treatment-naïve)	24	81,070	15.874	benchmark	
	Initial: Peginterferon + Ribavirin; Follow-up: Sofosbuvir + Ribavirin for non-responders and relapsers.	24 + 12	81,920	18.4461	330	330
		24 + 16	82,240	19.3293	339	362
	Sofosbuvir + Ribavirin (Treatment-naïve)	12	119,940	18.4217	15,257	dominated
3	Peginterferon + Ribavirin (Treatment-naïve)	24	121,170	12.2543	benchmark	
	Initial: Peginterferon + Ribavirin; Follow-up: Sofosbuvir + Ribavirin for non-responders and relapsers.	24 + 16	147,520	17.1869	5,342	5,342
		24 + 12	162,490	13.8563	25,793	dominated
	Sofosbuvir + Ribavirin (Treatment-naïve)	24	191,280	19.3615	9,865	20,123

For genotype 1 patients without cirrhosis, compared to the acknowledged efficient benchmark treatment (peginterferon + ribavirin + telaprevir), all new treatments are cost-effective with ICER less than the threshold of $50,000/QALY. In particular, treatments Harvoni and Viekira Pak both achieve higher QALYs with reduced costs, which makes the benchmark treatment no longer efficient, whereas treatments olysio + sovaldi and sofosbuvir + peginterferon + ribavirin both achieve higher QALYs but with increased costs compared to benchmark treatment. However, if compared to Viekira + Pak, Harvoni, olysio + sovaldi and sofosbuvir + peginterferon + ribavirin are no longer efficient characterized with higher costs and lower QALYs. Thus, we conclude that all four new regimens are alternatives of the current standard care of treatment (peginterferon + ribavirin + telaprevir), but Viekira Pak is the most cost-effective for genotype 1 patients without cirrhosis, whereas the other three sofosbuvir-based treatments are featured with higher costs and lower QALYs. For genotype 2 treatments, compared to standard care of treatment (peginterferon + ribavirin), all three sofosbuvir-based treatments are cost-effective. However, the comparative ICER of two-phase treatment with a 16-week follow-up versus 12-week single treatment of sofosbuvir + ribavirin is $1,805,952/QALY, which is far beyond the threshold. Thus, for genotype 2 the two-phase treatments with peginterferon + ribavirin as initial and 12 and 16 weeks of sofosbuvir as follow-up are cost-effective, whereas single treatment with sofosbuvir + ribavirin is not. For genotype 3, similarly, the two-phase sofosbuvir-based treatments are cost-effective compared to the standard care of treatment; note that ICERs between adjacent treatments are also below the threshold. The 24-week single treatment with sofosbuvir + ribavirin is not cost-effective compared to standard care of treatment with an ICER of $88,833/QALY. Further, it is not cost-effective compared to two-phase treatment with a 16-week follow-up with an ICER of $374,594/QALY. Overall, single-phase treatment with sofosbuvir for both genotype 2 and 3 patients dominates two-phase treatments using peginterferon + ribavirin regimen as initial and sofosbuvir-based new treatment as follow-up. The reason is attributable to the fact that the traditional regimen (peginterferon + ribavirin) has a relatively high SVR for genotypes 2 and 3, and the new treatment's additional SVR increase does not justify its much higher cost.

When patients with cirrhosis are considered, for genotype 1 all new treatments are efficient compared to the standard care of treatment, whereas adjacent ICERs show that sofosbuvir + peginterferon + ribavrin and Viekira Pak are both inefficient compared to Harvoni with higher costs and lower QALYs. Although olysio + sovaldi has higher cost as well as higher QALYs compared to Harvoni, the ICER is $1,305,213/QALY, which is far beyond the threshold and hence not efficient. Therefore, for genotype 2 patients with cirrhosis, Harvoni is the most cost-effective treatment. For genotype 2, both two-phase treatments with 12-week and 16-week follow-ups are cost-effective, whereas 12-week single treatment with sofosbuvir + ribavirin is not cost-effective. For genotype 3 treatments, a 24-week single treatment with sofosbuvir + ribavirin is cost-effective, as well as the two-phase treatment with a 16-week follow-up.

### Sensitivity analysis

One-way sensitivity analyses are performed on prices, transition probabilities, SVR rates, utility weights and costs of care. Only when the sofosbuvir price is reduced by at least 30 %, olysio + sovaldi and sofosbuvir + peginterferon + ribavirin can achieve cost-effectiveness compared to Harvoni or Viekira Pak for genotype 1 patients. However, even when the sofosbuvir price is halved, using sofosbuvir as initial treatment is still not cost-effective compared to two-phase treatments that use sofosbuvir as a follow-up treatment for genotypes 2 and 3. Further, increasing SVR for sofosbuvir-based treatments by 10 % does make them cost-effective compared to Harvoni and Viekira Pak for genotype 1 patients, and reducing SVR for Harvoni and Viekira Pak by 10 % also makes sofosbuvir-based treatments cost-effective.

Changing SVR rates of sofosbuvir-based treatments for genotypes 2 and 3 does not change the effectiveness of the treatments. Two-phase treatments of 24 + 12 weeks are always cost-effective for both genotypes 2 and 3, compared to which, a change in the values of cost of care or utility weights can push 24 + 16 weeks treatments’ ICER beyond or below $50,000/QALY when benchmarked with the base case. However, it does not change the conclusion that single phase treatment of sofosbuvir + ribavirin is not cost-effective for both genotypes 2 and 3. Thus, for genotype 1 patients, a reduction in sofosbuvir price can improve the cost-effectiveness of sofosbuvir-based treatments compared to the alternatives of Harvoni and Viekira Pak. For genotypes 2 and 3, however, sofosbuvir-based new treatments serve better as follow-up treatments rather than initial treatments.

**Fig. 1:**
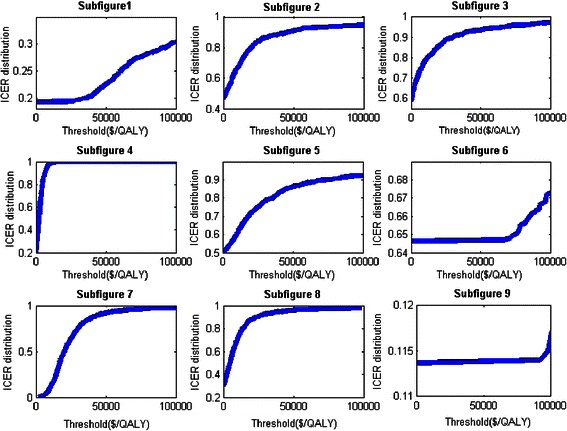
Cost-effectiveness Acceptability Curves between Treatments. Olysio + Sovaldi versus Harvoni for Genotype 1 (*Subfigure 1*); Viekira Pak versus Harvoni for Genotype 1 (*Subfigure 2*); Sofosbuvir + Peginterferon + Ribavirin versus Harvoni for Genotype 1 (*Subfigure 3*); Two-phase of 24 + 12 versus Peginterferon + Ribavirin for Genotype 2 (*Subfigure 4*); Two-phase of 24 + 16 versus Two-phase of 24 + 12 for Genotype 2 (*Subfigure 5*); Sofosbuvir + Ribavirin versus Two-phase of 24 + 16 for Genotype 2 (*Subfigure 6*); Two-phase of 24 + 12 versus Peginterferon + Ribavirin for Genotype 3 (*Subfigure 7*); Two-phase of 24 + 16 versus Two-phase of 24 + 12 for Genotype 3 (*Subfigure 8*); Sofosbuvir + Ribavirin versus Two-phase of 24 + 16 for Genotype 3 (*Subfigure 9*)

Probabilistic analysis results are shown via the cost-effectiveness acceptability curves (Fig. 1), which are interpreted as the probability that the data are consistent within a true cost-effectiveness ratio falling below that value between two treatments. The figure that the ICER of olysio + sovaldi versus Harvoni falls either below 0 or above $50,000/QALY with approximate probability 0.95, implying that compared to Harvoni, olysio + sovaldi either achieves lower QALYs with higher costs or achieves higher QALYs with higher cost but with ICER exceeding the threshold $50,000/QALY. Thus, with 95 % confidence we conclude that olysio + sovaldi is not cost-effective. For Viekira Pak and sofosbuvir + peginterferon + ribavirin, compared to Harvoni, they both have roughly a 0.50 probability of being effective according to figure. For both genotypes 2 and 3, the ICER of two-phase treatment with peginterferon + ribavirin as initial and 12-week sofosbuvir as follow-up versus the standard of treatment is below $10,000/QALY with 0.95 probability according to the figure, whereas two-phase treatment with peginterferon + ribavirin as initial, a 16-week sofosbuvir as follow-up and single treatment with sofosbuvir + ribavirin both have equal probability of being effective and not effective for genotypes 2 and 3.

## Discussion

Our analyses show that Viekira Pak is cost-effective for genotype 1 patients without cirrhosis, while Harvoni is cost-effective for genotype 1 patients with cirrhosis. Sofosbuvir-based treatments for genotype 1, in general, are not cost-effective due to its substantial high costs. The price of Sofosbuvir would need to be reduced by at least 30 % in order for olysio + sovaldi and sofosbuvir + peginterferon + ribavirin to achieve cost-effectiveness. For genotypes 2 and 3, sofosbuvir + ribavirin as initial treatment comes with a large increase in cost and a small increase in effectiveness. It is not recommended as an initial treatment for patients with genotypes 2 and 3, with the exception of genotype 3 with cirrhosis, in which case, a 24-week sofosbuvir + ribavirin treatment is cost-effective as it leads to much higher SVR compared to alternative treatments. As a second-phase treatment for genotypes 2 and 3, sofosbuvir + ribavirin is cost-effective following peginterferon + ribavirin as initial treatment.

To assure the robustness of our results, we performed sensitivity analyses over wide ranges for all model parameters, and the results did not significantly impact our final conclusions. However, it is important to note that the results obtained in this study should be interpreted within the model assumptions, and there are several limitations. First, model parameters were obtained from the literature and there is limited data on sofosbuvir-involved treatment. Further, the SVR rates used from the five trials targeted different patient cohorts. Bias in these past studies could impact the results presented here. In addition, the Markov model used in the analysis is a simplified representation of disease progression based on aggregate population transitions. Although we believe that we have captured the primary stages, a higher fidelity model could lead to different conclusions. Finally, the population data is specific to the United States, and so the results might not be directly applicable to other countries.

## Conclusions

We analyzed the cost-effectiveness of sofosbuvir-based new treatments for genotypes 1, 2 and 3 in the US. Data regarding the natural history of hepatitis C, utility weights, and various costs and transition probabilities were obtained from the literature, and sustained virologic response data associated with new treatments were extracted from clinical studies. Treatment strategies compared in this study were designed based on the data available while complying with drug dosage and administrative recommendations.

We found that for genotype 1 patients, sofosbuvir-based treatments are not cost-effective compared to Viekira Pak and Harvoni. If the price of sofosbuvir were reduced by at least 30 %, then this would change this result. In addition, Sofosbuvir + ribavirin are cost-effective as second-phase treatments following peginterferon + ribavirin initial treatment for genotypes 2 and 3. However, the data for sofosbuvir-involved treatment is limited. Therefore, the results obtained in this study must be interpreted within the model assumptions.

## Abbreviations

CHC: Chronic Hepatitis C
EVR: Earlier virologic response
FDA: US Food and Drug Administration
HCC: Hepatocellular carcinoma
LLOQ: Lower limit of quantification
QALY: Quality adjusted life year
SVR: Sustained virologic response
